# Protocol for isolation and flow cytometric analysis of interstitial macrophages in murine lung metastasis models

**DOI:** 10.1016/j.xpro.2025.104060

**Published:** 2025-09-07

**Authors:** Mi Reu Jeong, Seung Hyeok Seok

**Affiliations:** 1 Macrophage Lab, Department of Microbiology and Immunology, and Institute of Endemic Disease, Seoul National University College of Medicine, Seoul 03080, Republic of Korea; 2 Department of Biomedical Sciences, Seoul National University College of Medicine, Seoul 03080, Republic of Korea; 3 Cancer Research Institute, Seoul National University, Seoul 03080, Republic of Korea

**Keywords:** Cell Biology, Flow Cytometry, Cancer, Immunology

## Abstract

Interstitial macrophages increase significantly during lung metastasis and may contribute to tumor dissemination. However, isolating them is challenging due to their localization within lung tissue and phenotypic overlap with other immune cells. Here, we present a protocol for isolating and characterizing murine interstitial macrophages. We include steps for establishing lung metastasis models, lung tissue dissociation, fluorescent antibody staining, and flow cytometry analysis. It is applicable to both metastasized and naive lung tissues.

For complete details on the use and execution of this protocol, please refer to Jeong et al.^1^

## Highlights

• Steps for generating mouse lung metastasis models using cancer cell lines
• Procedures for isolating interstitial macrophages from metastasized lungs
• Guidance for gating lung interstitial macrophages for flow cytometry

## Abstract (editor highlights)

Publisher’s note: Undertaking any experimental protocol requires adherence to local institutional guidelines for laboratory safety and ethics.

## Graphical abstract

**Figure:**
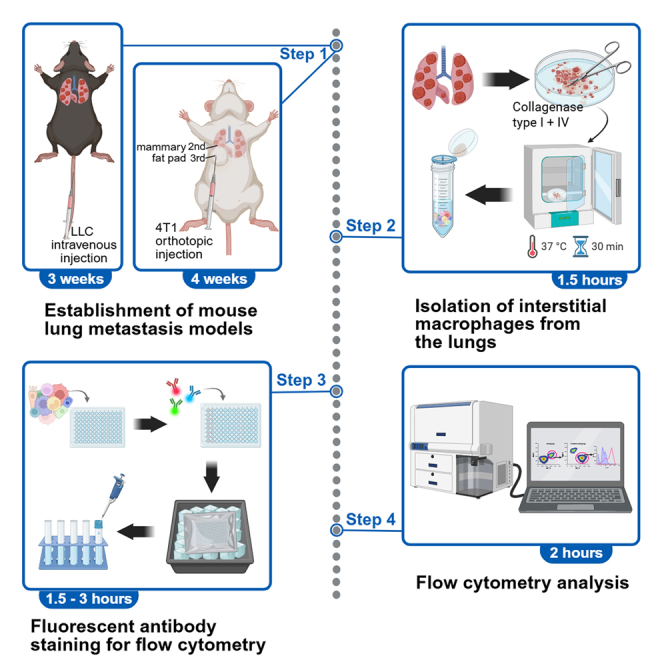

## Before you begin

The lungs are a common site of metastatic tumor colonization, and macrophages have been shown to play a critical role in promoting metastatic progression.^2^ Pulmonary macrophages are broadly classified into two subsets: alveolar macrophages, which are easily obtained through bronchoalveolar lavage (BAL) fluid sampling due to their location in the alveoli^3^; and interstitial macrophages, which are embedded within the lung parenchyma and are considerably more difficult to isolate.^4^ Interstitial macrophages are derived from circulating monocytes and share phenotypic markers with other mononuclear cells, complicating their identification and analysis.^5^ This protocol describes a detailed method for the isolation of interstitial macrophages from metastasized murine lungs, as well as procedures for cell preparation and flow cytometric analysis.

### Innovation

This protocol introduces a streamlined approach for generating lung metastasis models and provides practical, hands-on guidance for dissociating lung tissues without the need for specialized equipment. A key advancement is the ability to clearly distinguish interstitial macrophages from other lung mononuclear cells using refined gating strategies and more specific surface markers, reducing the ambiguity often encountered in immunophenotyping. By integrating these methodological improvements into a unified workflow, this protocol enhances both reproducibility and accessibility for a broad range of laboratories.

### Institutional permissions

All animal experiments were performed in accordance with the guidelines of the Institute of Laboratory Animal Resources and the Institutional Animal Care and Use Committee (IACUC) at Seoul National University (IACUC numbers: SNU-221126-1-6 and SNU-210818-1-7).

### Preparation of cancer cells

**Timing: 2 weeks**

1. Thawing cancer cells (Lewis lung carcinoma (LLC) or 4T1 cell lines).
  a. Thaw 1.5–2 × 10^6^ cells/1 mL of cryopreserved cell (5% DMSO and 95% fetal bovine serum [FBS] in a 37°C water bath.
  b. Transfer the thawed cells into 9 mL culture medium in a 15 mL tube.
  c. Centrifuge at 300 *g* for 5 min and remove medium containing DMSO.
2. Seeding cancer cells.
  a. Add 15 mL culture medium into a T-75 flask.
  b. Resuspend the cell pellet in 1 mL of culture medium.
  c. Seed the cells at a density of 1.5 × 10^6^ cells per T-75 flask.
    i. Prepare 27 μL of 0.2% trypan blue (diluted with 1X PBS) in a 1.5 mL tube.
    ii. Take 3 μL of the cells and mix with the trypan blue solution.
    iii. Place 10 μL of the cell suspension into the counting chamber and count cells in at least two corner squares or in the center squares of the grid.
      ***Note:*** If cells lie on the grid lines, count only those touching the top and left borders or the bottom and right borders, but be consistent throughout the count.
    iv. Divide the total number of cells counted by the number of squares used (e.g., corner or center squares). And multiply the result by 10^5^ to account for the 10-fold dilution with trypan blue.
      ***Note:*** A cell suspension viability (trypan blue-negative cells) of over 80% generally ensures healthy cell growth.
  d. Incubate the cells in a 5% CO_2_, 37°C cell incubator for 2 days.
3. Cancer cell subculture.
  a. Aspirate the culture medium.
    ***Note:*** For LLC, floating cells are also viable; thus, culture medium can be collected and centrifuged to yield a higher number of cells if needed.
  b. Wash the cells once with 10 mL of sterile 1X PBS.
  c. Add 1 mL of trypsin-EDTA and spread thoroughly to cover all cells.
  d. Incubate the cells in a 5% CO_2_, 37°C cell incubator.
    i. 2 min for LLC cell line.
    ii. 5 min for 4T1 cell line.
  e. Add 9 mL of culture medium and transfer the cells into a 15 mL tube.
    **CRITICAL:** Ensure all cells are detached and resuspended from the flask.
  f. Centrifuge at 300 *g* for 5 min.
  g. Place 15 mL culture medium into T-75 flask.
  h. Resuspend the cells with 1 mL culture medium.
  i. Seed the cells at a density of 1.5 × 10^6^ cells viable cells per T-75 flask, ensuring viability is above 80%.
  j. Incubate the cells in a 5% CO_2_, 37°C cell incubator for 2 days.
    **CRITICAL:** Subculture at least twice before injecting into mice. Check cell lines for mycoplasma contamination using a mycoplasma PCR detection kit.
4. Preparation of cell suspension.
  a. Aspirate the culture medium.
  b. Wash the cells once with 10 mL of sterile 1X PBS.
  c. Add 1 mL of trypsin-EDTA and spread thoroughly to cover all cells.
  d. Incubate the cells in a 5% CO_2_, 37°C cell incubator for 2 min for LLC and 5 min for 4T1.
  e. Add 9 mL of culture medium and transfer the cells into a 15 mL tube.
  f. Centrifuge at 300 *g* for 5 min at 4°C.
  g. Aspirate the medium and add 1 mL of ice-cold 1X PBS.
  h. Add an additional 9 mL of ice-cold 1X PBS to the cells in the 15 mL tube.
  i. Centrifuge at 300 *g* for 5 min at 4°C.
    **CRITICAL:** Ensure trypan blue-negative (live) cells >95%.
  j. Resuspend the cells at a density of 5 × 10^5^ cells/200 μL for LLC and 1 × 10^6^ cells/100 μL for 4T1 in ice-cold 1X PBS.
  k. Aliquot the 1 mL of the cell suspension into 1.5 mL tubes.
  l. Keep the cells on ice.
    **CRITICAL:** Ensure that cells are not left on ice for more than 2 h.

### Generation of a murine lung metastasis model via intravenous injection

**Timing: 3 weeks**

**Figure 1:**
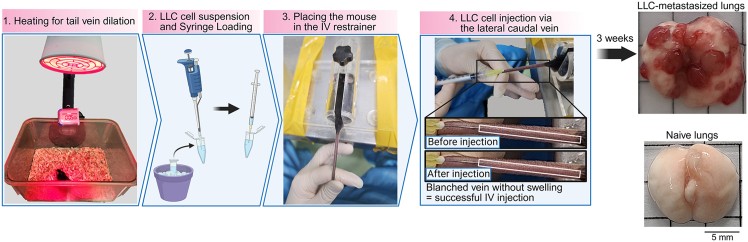
Intravenous injection of cancer cells Scale bar, 5mm.

***Note:*** This will help dilate the tail veins.

5. Prepare mice for injection (Figure 1).
  a. Place the mouse under an infrared heat lamp for about 5 min.

**CRITICAL:** Use C57BL/6 mice for LLC cell injection.

6. Secure the injection setup (Figure 1).
  a. Secure the mouse IV injection restrainer on the desk using tape.
7. Prepare the cell suspension (Figure 1).
  a. Resuspend the cells using a 1 mL pipette.
  b. Load 200 μL of the cancer cell suspension into a syringe.
    i. Use a 100-unit insulin syringe or a 1 mL syringe with a 30-gauge needle.

**CRITICAL:** Avoid air bubbles.

8. Inject cancer cells (Figure 1).
  a. Place the mouse in the IV restrainer.
  b. Hold the tail and rotate it approximately 90° to the left or right.
    i. Position the lateral caudal veins facing upward; this is where the injection will be made.
  c. Use alcohol to disinfect the tail surface.
    ***Note:*** This will also help visualize the lateral caudal veins more clearly in C57BL/6 mice.
  d. Use your thumb and second fingers to hold the tail straight. Use your third and fourth fingers to press against the desk to stabilize the tail and prevent movement.
  e. Insert the needle straight into the tail vein and slowly inject the cell suspension.
    i. Gently pull back the syringe plunger to check for blood return. This confirms that the needle is properly positioned in the vein.
      **CRITICAL:** If the needle is properly inserted, the cells will inject smoothly. If you feel resistance, the needle may not be in the vein—do not proceed. Secure the needle in place and avoid any movement during injection.
9. Wait approximately 3 weeks for the cancer cells to metastasize to the lungs (Figure 1).

***Note:*** Monitor the mice for any complications and conduct special daily monitoring beginning 2 weeks after the cancer cell injection.

### Generation of a murine lung metastasis model via orthotopic injection into the mammary fat pad

**Timing: 4 weeks**

**Figure 2:**
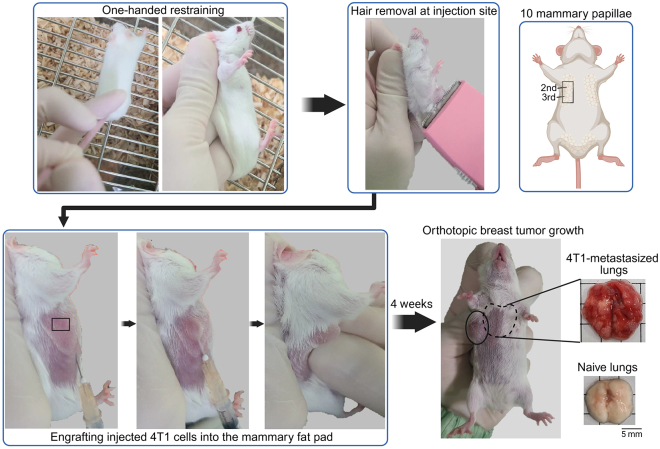
Orthotopic injection of cancer cells into the mammary fat pad Scale bar, 5mm.

10. Prepare the mouse for injection (Figure 2).
  a. Identify the 10 mammary papillae on the female mouse.
  b. Shave the hair on one upper limb.
    i. There are three papillae located on the upper side of each limb.

**CRITICAL:** Use BALB/c mice for 4T1 cell injection.

11. Prepare the cancer cell suspension.
  a. Resuspend the cells thoroughly using a 1 mL pipette.
  b. Load 100 μL of the cancer cell suspension into a 1 mL syringe.

**CRITICAL:** Avoid introducing air bubbles into the syringe.

12. Secure the mouse for injection (Figure 2).
  a. Use the little finger to hold the base of the tail and gently press along the back of the mouse, from the lower back to the upper back, to restrain it. Then, grasp the scruff of the neck, close to the ears.
  b. Gently raise the shaved upper limb on the injection side to expose the injection area.
13. Inject the cancer cells (Figure 2).
  a. Insert the needle horizontally under the skin, approximately one needle length away from the 2nd or 3rd mammary papilla and guide it toward the papilla.
  b. Slowly inject the cancer cell suspension.
  c. After injecting 4T1 cells into the mammary fat pad, apply gentle manual pressure to the injection site to aid in cell engraftment and prevent leakage.

***Note:*** You may feel slight resistance when inserting the needle toward the mammary papilla—this indicates that the needle is in the mammary fat pad.

14. Allow 4 weeks for metastasis to develop in the lungs (Figure 2).

***Note:*** Monitor the mice for complications and begin daily monitoring starting at 2 weeks after cancer cell injection.

## Key resources table

**Table:** 

REAGENT or RESOURCE	SOURCE	IDENTIFIER
**Antibodies**		

Anti-mouse CD45 Alexa Fluor 700 (I3/2.3) (working concentration: 2.5 μg/mL)	BioLegend	Cat# 147716; RRID:AB_2750449
Anti-mouse CD11b eFluor 450 (M1/70) (1 μg/mL)	Thermo Fisher Scientific	Cat# 48-0112-82; RRID:AB_1582236
Anti-mouse Ly6C BV605 (HK1.4) (0.25 μg/mL)	BioLegend	Cat# 128036; RRID:AB_2562353
Anti-mouse Ly6G FITC (1A8) (2.5 μg/mL)	BioLegend	Cat# 127606; RRID:AB_1236494
Anti-mouse CD3 FITC (C363.29B) (2.5 μg/mL)	Southern Biotech	Cat# 1535-02, RRID:AB_2794819
Anti-mouse CD64 PerCP-eFluor 710 (X54.5/7.1) (2.5 μg/mL)	Thermo Fisher Scientific	Cat# 46-0641-82; RRID:AB_2735016
Anti-mouse MERTK Super Bright 780 (DS5MMER) (2.5 μg/mL)	Thermo Fisher Scientific	Cat# 78-5751-82; RRID:AB_2762814
Anti-mouse MHC class II (I-A/I-E) APC-eFluor 780 (M5/114.15.2) (2.5 μg/mL)	Thermo Fisher Scientific	Cat# 47-5321-82; RRID:AB_1548783
Anti-mouse Siglec F BV421 (E50-2440) (2.5 μg/mL)	BD Biosciences	Cat# 562681; RRID:AB_2722581
Anti-mouse/human Arginase 1 APC (A1exF5) (2.5 μg/mL)	Thermo Fisher Scientific	Cat# 17-3697-82; RRID:AB_2734835
Anti-mouse purified CD16/32 antibody (93) (2.5 μg/mL)	BioLegend	Cat# 101302; RRID:AB_312801

**Biological samples**		

Mouse tissue samples (lungs)	Seoul National University Institutional Animal Care and Use Committee	IACUC#: SNU-210818-17 SNU-221126-1-6;

**Chemicals, peptides, and recombinant proteins**		

10X Hank’s balanced salt solution (HBSS)	WELGENE	LB 203-06
Dulbecco’s modified Eagle’s medium (DMEM)	WELGENE	LB 001-05
Roswell Park Memorial Institute Medium (RPMI) 1640	WELGENE	LM 011-01
Bovine serum albumin (BSA)	Bovogen	Cat# BSAS 0.1
20X Phosphate Buffered Saline (PBS)	iNtRON Biotechnology	Cat# IBS-BP007
Deoxyribonuclease I	Sigma-Aldrich	Cat# D5025-150KU
Collagenase, type I	Sigma-Aldrich	Cat# C0130
Collagenase, type IV	Sigma-Aldrich	Cat# C5138
Zombie Aqua fixable viability kit	BioLegend	Cat# 423102
IC Fixation buffer	Thermo Fisher Scientific	Cat# 00-8222-49
0.05% Trypsin-EDTA (1X)	Gibco	Cat# 25300-054
Penicillin Streptomycin	Gibco	Cat# 15140-122
RNAiso Plus	Takara	Cat# 9109
BD Horizon Brilliant Stain Buffer Plus	BD Biosciences	Cat# 566385
Myco-Sniff Mycoplasma PCR Detection Kit	MP Biomedicals	Cat# 093050201

**Experimental models: Cell lines**		

LLC	Wenes et al.^6^	N/A
4T1	ATCC	Cat# CRL-2539; RRID:CVCL_0125

**Experimental models: Organisms/strains**		

C57BL/6 wild-type male mice, 6–12 weeks old	Orient Bio	N/A
BALB/c wild-type female mice, 6–12 weeks old	Orient Bio	N/A

**Others**		

5 mL tube	Watson	Cat# 233-150C
5 mL tube, screw cap	Eppendorf	Cat# 0030122313
Iris Scissors straight	KASCO	Cat# 5-005
Iris Scissors curved	Hebu	Cat# HB 7459
Iris Forcep straight	KASCO	Cat# 50-2000
30-gauge needle, 13 mm	Sungshim	30GX13mm
1 mL syringe	Sungshim	HJ-1 26X13mm
1 mL insulin syringe, 0.3 mm × 8 mm	Sungshim	1mL/cc 30G × 8mm
10 mL syringe	Sungshim	KOVAX-SYRINGE 10mL 22G 11/4″
500 mL vacuum filtration flask	Nest	Cat# 343021
T-75 cell culture flask	SPL	Cat# 70075
Counting chamber	Marienfeld Superior	Cat# HSU-0650030

## Materials and equipment

**Table:** 1X HBSS solution

Reagent	Final concentration	Amount
10X HBSS	1X	100 mL
Penicillin Streptomycin	1%	10 mL
ddH_2_O	N/A	890 mL
**Total**	**N/A**	**1 L**

Filter the solution through a 0.22 μm bottle-top filter and store at 4°C. Use within six months.

**Table:** DNase I solution

Reagent	Final concentration	Amount
0.15 M NaCl	150 mM	2 mL
DNase I	150,000 UNITS	∼94 mg
**Total**	**N/A**	**2 mL**

Aliquot approximately 50 μL of the solution into 1.5 mL tubes and store at −20°C for up to 1 year.

**Table:** Collagenase solution (4 mL per lungs)

Reagent	Final concentration	Amount
Collagenase I	0.5 mg/mL	12 mg
Collagenase IV	0.5 mg/mL	12 mg
DNase I solution	75 units/mL (0.05 mg/mL)	24 μL
FBS	1%	240 μL
1X HBSS solution	N/A	<23.736 mL
**Total**	**N/A**	**24 mL**

Use immediately after preparation.

**Table:** FACS buffer

Reagent	Final concentration	Amount
1X PBS	1X	<500 mL
BSA	1.0%	5.0 g
Sodium azide	0.1%	0.5 g
**Total**	**N/A**	**500 mL**

Filter the solution through a 0.22 μm bottle-top filter and store at 4°C. Use within six months.

**Table:** MACS buffer

Reagent	Final concentration	Amount
1X PBS	1X	<498 mL
BSA	0.5%	2.5 g
0.5 M EDTA	2 mM	2 mL
**Total**	**N/A**	**500 mL**

Filter the solution through a 0.22 μm bottle-top filter and store at 4°C. Use within six months.

**Table:** Ammonium-chloride-potassium (ACK) buffer

Reagent	Final concentration	Amount
NH_4_Cl	150 mM	4 g
KHCO_3_	10 mM	0.5 g
0.5 M EDTA	0.1 mM	100 μL
ddH_2_O	N/A	<499.9 mL
**Total**	**N/A**	**500 mL**

Filter the solution through a 0.22 μm bottle-top filter and store at 4°C. Use within six months.

**Table:** Cell culture medium

Reagent	Final concentration	Amount
RPMI 1640 medium	1X	445 mL
Penicillin Streptomycin	1%	5 mL
FBS	10%	50 mL
**Total**	**N/A**	**500 mL**

Store at 4°C and use within six months.

## Step-by-step method details

### Interstitial macrophage isolation from metastasized lungs

**Timing: 1–2 h**

These steps enable the isolation of interstitial macrophages from highly metastasized lungs, which exhibit significant infiltration of myeloid cells. A higher metastatic burden typically yields a greater number of interstitial macrophages.

1. Euthanize the mice using appropriate methods.

***Note:*** A drop jar method using isoflurane or an overdose of chemical anesthetics (2–3 times the normal anesthetic dose) administered to one mouse at a time is preferable.

2. Perfuse the lungs.
  a. Fill a 10 mL syringe with PBS and replace the needle with a 26-gauge needle.
  b. Insert another 26-gauge needle into the inferior vena cava (under the liver) to create an outlet for blood during lung perfusion.
  c. Insert the PBS-filled syringe into the right ventricle to perform the perfusion.
3. Place the lungs in HBSS solution in a 5 mL tube and keep on ice.
4. Dissociate the lungs into single cells (Figure 3).
  a. Add 500 μL of collagenase solution to a 60 × 75 mm Petri dish.
  b. Place the lungs on the dry side of a Petri dish by slightly tilting the dish to allow the liquid to pool on one side. Using curved scissors, mince the tissue while keeping the cut pieces on the dry side.
    ***Note:*** This setup facilitates more thorough and efficient tissue mincing, allowing the minced tissue to be easily pipetted using 1000 μL pipette tips.
  c. Add an additional 3.5 mL of collagenase solution and pipette thoroughly using 1000 μL to ensure complete resuspension.
  d. Incubate the lungs at 37°C for 30 minutes.
    i. At the 15-minute mark, pipette the cell mixture again to facilitate tissue dissociation, ensuring it can be easily pipetted through 200 μL pipette tips.

**Figure 3:**
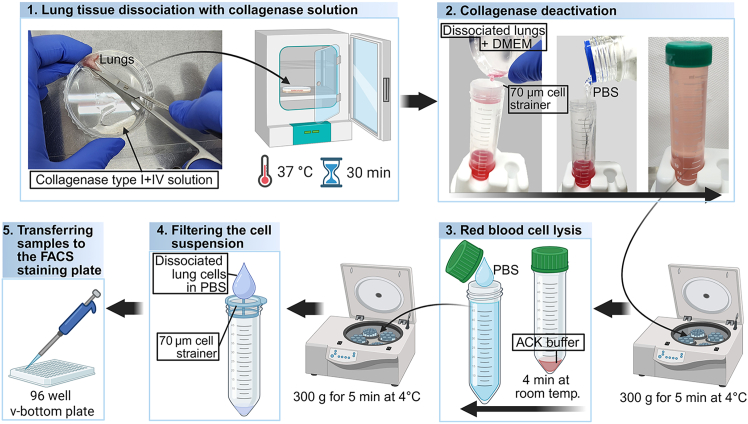
Schematic overview of the protocol used to dissociate lung tissue into a single-cell suspension
5. Inactivate the collagenase (Figure 3).
  a. Add cold DMEM or RPMI (with 10% FBS) at twice the digestion volume (8 mL).

***Optional:*** Add 0.5 mM EDTA to the solution.

6. Filter the cell suspension through a 70 μm nylon mesh filter into a 50 mL conical tube (Figure 3).
  a. Add PBS to bring the volume up to 50 mL after filtering.
7. Centrifuge at 300 *g* for 5 min at 4°C.
8. Aspirate the supernatant.
9. Resuspend the pellet in 1 mL of ACK buffer, then add 3 mL of additional ACK buffer and incubate for 4 min at room temperature (Figure 3).

***Note:*** Warm the ACK buffer to 36°C before use.

10. Add PBS to the cell suspension containing ACK buffer to bring the total volume to 50 mL (Figure 3).
11. Centrifuge at 300 *g* for 5 min at 4°C.
12. Aspirate the supernatant. Resuspend the pellet in 1 mL of PBS.
13. Filter the cells again through a 70.0 μm nylon membrane filter into a 50 mL tube (Figure 3).

***Note:*** The cells can be collected in a 1.5 mL tube by carefully filtering the suspension through a membrane filter to avoid spillage.

**CRITICAL:** To improve antibody staining efficiency, filter the cell suspensions to obtain a single-cell population, as tumor cells are particularly prone to clumping.

### Multiparameter flow cytometry for interstitial macrophage profiling

**Timing: 1 h**

These steps enable optimal staining and flow cytometric analysis of immune cells, with a specific focus on interstitial macrophages isolated from myeloid cell-rich, metastasized lungs. A multicolor antibody panel is used, carefully designed to distinguish interstitial macrophages with minimal overlap from phenotypically similar immune populations. This protocol also includes a comparative evaluation of commonly used marker combinations for the identification of interstitial macrophages.

14. Aliquot the cells into the wells of a 96 well v-bottom plate (Figure 3).
  a. Use 1 × 10^6^ to 5 × 10^6^ cells per well for flow cytometry staining.
15. Prepare samples for unstained, single-stained, and fully stained conditions.
  a. Prepare samples for unstained and single-stained controls by mixing aliquots from all samples.
  b. The number of wells used for single stains should match the number of dyes used in the experiment.
    i. Use the same antibody working concentration as that used for the fully stained samples.
16. Centrifuge 300 *g* for 5 min at 4°C.

**CRITICAL:** Always balance the plate before centrifugation.

***Note:*** To remove the supernatant after centrifugation, quickly flick the plate over a sink and gently tap it on a paper towel.

17. Stain cells and single-stained control for viability with 100 μL of Live/Dead dye for 15 min at room temperature.
  a. Dilute Live/Dead staining dye at 1:500 to 1:1000 with PBS.
  b. Use a multi-channel pipette to resuspend the cells.
  c. Protect from light by wrapping the plate in aluminum foil.

**CRITICAL:** From this point on, always protect the cells from light by wrapping the plate in aluminum foil during incubation.

**Figure 4:**
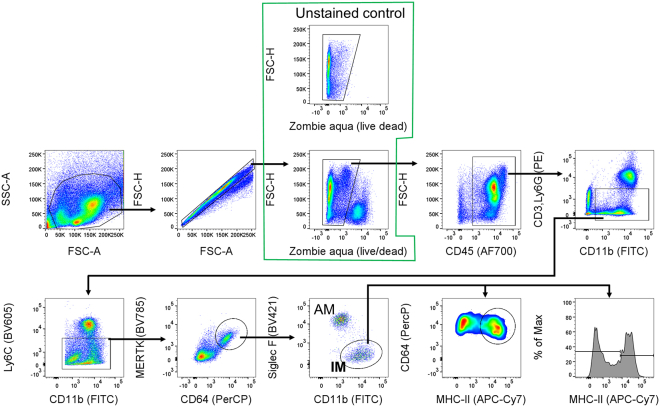
Flow cytometry gating strategies for lung interstitial macrophages

**Figure 5:**
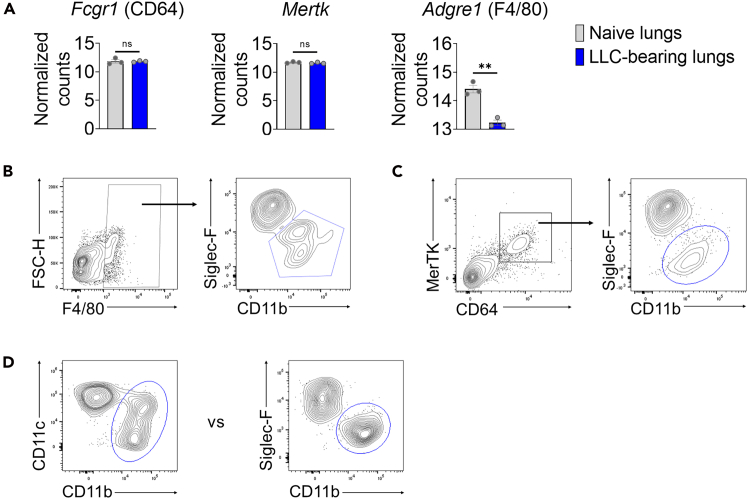
Macrophage markers in the lungs (A) Expression of macrophage-associated genes in interstitial macrophages isolated from naive and LLC-bearing lungs. Two-sample, two-sided t-tests were used (n = 3). All bars represent the mean ±SEM. ns = *p* > 0.05, ∗∗*p* < 0.01. (B) Contour plots showing interstitial macrophage populations gated on F4/80^+^ cells. (C) Contour plots showing interstitial macrophage populations gated on CD64^+^MerTK^+^ cells. (D) Contour plots comparing CD11c and Siglec-F expression for distinguishing alveolar macrophages from interstitial macrophages.
  g. Plot CD11b vs. Siglec-F to distinguish macrophage subtypes: Interstitial macrophages: CD11b^+^ Siglec-F^−^; Alveolar macrophages: CD11b^−^Siglec-F^+^.
    **CRITICAL:** CD11c is frequently used to identify alveolar macrophages; however, certain subsets of interstitial macrophages also express CD11c (Figure 5D). Consequently, CD11c expression may not clearly distinguish between alveolar and interstitial macrophage populations.
  h. Analyze MHC-II expression in interstitial macrophages using a histogram or contour/pseudocolor-smooth plot.
    ***Note:*** Since interstitial macrophages are present at low frequency in the lung, contour or smoothed pseudocolor plots are recommended for improved visualization.

18. Centrifuge at 300 *g* for 5 min at 4°C.
19. Wash the cells with 200 μL of FACS buffer.
20. Centrifuge again at 300 *g* for 5 min at 4°C.
21. Block Fc receptors using 50 μL of CD16/32 antibody for 15 min on ice.
  a. Dilute the CD16/32 antibody to 1 μg/mL in FACS buffer.
  b. Resuspend the cells using a multi-channel pipette.
22. Centrifuge 300 *g* for 5 min at 4°C.
23. Stain surface markers for 30 mins on ice in the dark.
  a. Dilute conjugated antibodies for surface markers to the appropriate working concentration in FACS buffer.
  b. If using multiple Brilliant Violet, or Super Bright dyes, mix 10 μL of Brilliant Stain Buffer Plus per test in the FACS buffer containing all antibodies, for a total volume of 50 μL per well.
24. Centrifuge 300 *g* for 5 min at 4°C.
25. Wash cells with 200 μL of FACS buffer.
26. Centrifuge again at 300 *g* for 5 min at 4°C.
27. For intracellular staining (If applicable).
  a. Fix the cells by adding 200 μL of IC Fixation Buffer.
  b. Incubate for 30 min on ice in the dark.
  c. Centrifuge at 300 *g* for 5 min at 4°C.
  d. Wash cells with 200 μL of 1X permeabilization buffer.
  e. Centrifuge at 300 *g* for 5 min at 4°C.
  f. Stain intracellular antigens for 30 min on ice in the dark.
    i. Dilute the antibodies to their working concentrations in 1X permeabilization buffer.
  g. Centrifuge at 300 *g* for 5 min at 4°C.
  h. Wash cells with 200 μL of 1X permeabilization buffer.
  i. Resuspend cells in 200 μL of FACS buffer.
  j. Filter the cells using 70 μm cell strainer.
  k. Transfer the cells to FACS tubes and add an additional 100 μL of FACS buffer.
28. FACS machine running.
  a. Prime the FACS machine (BD LSRFortessa X-20 or FACSymphony A3) to ensure there is no clogging.
  b. Set the parameters.
    i. Under FSC and SSC parameters, enable Height (H) and Width (W), to allow for the exclusion of doublets.
    ii. Use the violet (405 nm) laser for Zombie Aqua (BioLegend) Live/Dead staining, eFluor 450-conjugated CD11b antibody, BV605-conjugated Ly6C antibody, SB780-conjugated MerTK antibody, and BV421-conjugated Siglec-F antibody.
    iii. Use the blue (488 nm) laser for FITC-conjugated Ly6G/CD3 antibodies, and PerCP-conjugated CD64 antibody.
    iv. Use the red (637 nm) laser for Alexa Fluor 700-conjugated CD45 antibody, and APC-eFluor 780-conjugated MHC-II antibody.
  c. Record events for 300,000 live cells.
    i. Filter the sample again before recording to prevent clogging and ensure proper flow.
    ii. Set the x-axis to “Time” on a plot to monitor consistent flow through the FACS machine.
      **CRITICAL:** Consistent cell flow without clogging is essential. Use the time plot to check for irregularities. Also, avoid introducing air bubbles into the machine.
  d. Export the data as FCS files.
    ***Note:*** Label all sample files clearly and consistently.
29. Analyze the data using FlowJo and generate flow cytometry plots (Figure 4).
  a. Gate FSC-A (x-axis) vs. FSC-H (y-axis) to exclude doublets.
  b. Gate live cells to identify viable populations.
    i. Use either unstained controls or Fluorescence Minus One (FMO) controls to accurately gate the live cell population.
  c. Gate CD45 (x-axis) vs FSC-H (y-axis) to select CD45^+^ leukocytes.
  d. Plot CD11b (x-axis) vs. Ly6G/CD3 (y-axis) to exclude neutrophils and lymphocytes by gating on Ly6G^−^/CD3^−^ cells.
  e. Plot CD11b (x-axis) versus Ly6C (y-axis) to exclude monocyte populations.
  f. Gate on CD64^+^ MerTK^+^ double-positive cells to identify pan-macrophages in the lung.
    **CRITICAL:** F4/80 is commonly used as a macrophage marker; however, it has limitations in this context. Dendritic cells and eosinophils can also express F4/80, and its transcriptomic expression in macrophages may fluctuate depending on the inflammatory state or lung pathology (Figure 5A). In contrast, the transcriptomic expression of CD64 and MerTK is more stable in LLC-bearing lungs (Figure 5A). When comparing gating strategies, F4/80 does not clearly distinguish alveolar macrophages from interstitial macrophages (Figure 5B). In comparison, gating based on CD64^+^MerTK^+^ expression provides a clearer separation and enhanced resolution of macrophage subsets (Figure 5C). Therefore, we recommend using CD64^+^MerTK^+^ as a more reliable and specific pan-macrophage gating strategy.

## Expected outcomes

Numbers of interstitial macrophages per lungs are about 7-8 X 10^4^ cells in 7 weeks to 10 weeks naive C57BL/6 male mice. And 3.6–5 X 10^5^ cells in LLC-bearing mice. The use of CD45^+^Ly6G^−^Ly6C^−^CD64^+^MerTK^+^Siglec-F^-^CD11b^+^ markers enables clear and distinct flow cytometric gating of interstitial macrophages in both naive and metastasized lungs. This strategy allows for accurate isolation of interstitial macrophages, even in environments with heavy myeloid cell infiltration, thereby improving the precision of downstream analyses such as RNA sequencing.

## Limitations

Cell lines were used to generate murine lung metastasis models, which may not fully replicate the characteristics of spontaneous metastasis models. Interstitial macrophages make up less than 0.5% of total cells in naive lungs, and as such, their detection and characterization in other lung metastasis models may vary.

## Troubleshooting

### Problem 1

Presence of undigested lung tissue fragments after collagenase incubation.

### Potential solution

• Always prepare a fresh collagenase solution immediately before use to ensure enzymatic activity.
• During incubation, pipette the suspension frequently to enhance mechanical dissociation.

### Problem 2

Poor FACS staining quality.

### Potential solution

• Perform an additional round of red blood cell lysis using ACK buffer to eliminate residual erythrocytes that may interfere with antibody staining.
• Ensure the cell suspension is free of clumps prior to staining, as aggregates can obstruct uniform antibody access and impact staining quality.
• Incubate samples on ice for more than 30 min but less than 1 hour, protected from light, to enhance staining consistency.
• For optimal staining results, use approximately 1 × 10^6^ to 5 × 10^6^ cells per well.

### Problem 3

FACS plots appear distorted.

### Potential solution

• Filter the cell suspension immediately before FACS acquisition, particularly when working with tumor cells, which have a high tendency to clump.
• If needed, fix the cells using IC Fixation Buffer (Thermo Fisher) on ice for 20 min prior to FACS acquisition to enhance sample stability and minimize clumping caused by the adhesive properties of macrophages.
• Open the FCS file in FlowJo, and in the compensation matrix, set all values to zero. Adjust the numbers as needed to correct for distortion and optimize the figure display.

### Problem 4

The frequency of interstitial macrophages is low (<0.2% of the total lung cells) in flow cytometry analysis.

### Potential solution

• High levels of metastasis in the lungs (Figure 1) are necessary to yield a significant population of interstitial macrophages.
• During perfusion, slowly and gently inject PBS to minimize tissue disruption.
• Myeloid cell populations (CD11b^+^) can overlap with interstitial macrophages, including neutrophils (CD11b^+^Ly6G^+^) and monocytes (CD11b^+^Ly6C^+^), which are major contributors to cell accumulation during lung metastasis. Therefore, gating on cells negative for neutrophil and monocyte markers will more accurately identify and distinguish interstitial macrophages in flow cytometry plots.
• Perform FACS acquisition on the same day as lung digestion and staining to ensure optimal data quality.

## Resource availability

### Lead contact

Further information and requests for resources and reagents should be directed to and will be fulfilled by the lead contact, Seung Hyeok Seok (lamseok@snu.ac.kr).

### Technical contact

Technical questions on executing this protocol should be directed to and will be answered by the technical contact, Mi Reu Jeong (legulusidus@snu.ac.kr).

### Materials availability

This protocol does not involve any new or unique materials.

### Data and code availability

The transcriptomic sequencing data have been deposited to Dryad: https://doi.org/10.5061/dryad.jm63xsjjc.

## Acknowledgments

We thank the Flow Cytometry Core Facility at Seoul National University Hospital for their assistance with interstitial macrophage sorting. We also thank Professor Massimiliano Mazzone (KU Leuven, Belgium) for providing the metastasizing LLC cell line. This research was supported by a National Research Foundation of Korea (NRF) grant funded by the Korean government (MSIT) (RS-2024-00356146). It was also supported under the framework of the international cooperation program managed by the NRF (RS-2023-NR121106).

## Author contributions

Conceptualization, M.R.J. and S.H.S.; formal analysis, M.R.J.; methodology, M.R.J.; investigation, M.R.J.; visualization, M.R.J.; writing – original draft, M.R.J.; writing – review & editing, M.R.J. and S.H.S.; funding acquisition, S.H.S.

## Declaration of interests

The authors declare no competing interests.
